# The Impact of Low-Level Viraemia on Virological Failure—Results From a Multicenter HIV Antiretroviral Therapy Cohort Study in Yunnan, China

**DOI:** 10.3389/fmed.2022.939261

**Published:** 2022-07-04

**Authors:** Jing An, Yunfei Lao, Songyuan Tang, Jincheng Lou, Tianshu Li, Xingqi Dong

**Affiliations:** ^1^AIDS Clinical Management Office, Yunnan Provincial Infectious Disease Hospital, Kunming, China; ^2^Public Health School, Kunming Medical University, Kunming, China

**Keywords:** HIV, antiretroviral therapy, low-level viraemia, virological failure, Yunnan

## Abstract

**Background:**

HIV viral load (VL) is an important indicator to monitor treatment response in antiretroviral therapy (ART). Patients on ART may experience viral blips, with low-level elevations of VL between 50 and 999 copies/mL known as low-level viraemia (LLV), but not reaching the threshold for virological failure (≥1,000 copies/mL) defined by WHO guidelines. The objective was to investigate the long-term impact of LLV on virological failure.

**Methods:**

We analyzed adults who were ART naïve at baseline. LLV was defined as having an VL of 51–999 copies/mL at least once. The subjects with LLV were grouped into three categories: 51–199, 200–399, and 400–999 copies/mL. Patients with multiple episodes of LLV were classified based on the highest VL result. The subjects with LLV were also grouped by the frequency of LLV, i.e., a single episode, two consecutive episodes, two intermittent episodes, more than two consecutive episodes, and more than two intermittent episodes. Multivariable Cox models were used to predict the association of LLV with virological failure.

**Results:**

A total of 93,944 subjects were included. The median number of VL tests performed was 3. There were 21,203 LLV cases, with an overall incidence of 22.6%. Most of the LLV cases were found in subjects with LVs of 50–199 copies/mL, followed by 400–999 and 200–399 copies/mL. Most of the LLV cases experienced single episodes, and the numbers of LLV with two consecutive episodes, two intermittent episodes, more than two consecutive episodes and more than two intermittent episodes were decreased successively. The risk factors associated with virological failure include: intermediate-level (200–399 copies/mL) and high-level (400–999 copies/mL) LLV, single episodes of LLV and two or more than two consecutive episodes of LLV, which may put the subjects at a 1.28–2.26-fold higher risk for virological failure.

**Conclusion:**

Strengthened immediate medical attention should be placed on patients with VL of 200–999 copies/mL. The patients having experienced LLV once should be targeted for case management and repeat VL testing within 24 weeks to determine persistent LLV and monitor virological failure.

## Introduction

The invention of antiretroviral therapy (ART) in 1996 led to significantly improved quality of life for persons infected with the human immunodeficiency virus (HIV) in the long term. Although ART cannot completely cure AIDS, it helps to suppress viral load (VL), maintain functions of the immune system and prevent disease progression to AIDS or death (1). At the same time, virological suppression hinders the development of HIV resistance to antiretroviral drugs and minimizes HIV transmission (2, 3). One of the purposes of ART is to maximize inhabitation of viral replication suppression and reduce viral load to the lower limits of detection (LLOD) (4, 5). Introduction and use of more sensitive diagnostics resulted in decreased LLOD, i.e., from <400 copies/mL in first-generation assay to <50 copies/mL, and then to <20 copies/mL most recently (4–6).

Although optimal viral load suppression below LLOD is expected, some patients may experience virological failure (VF) or persistent Low-level Viraemia (LLV), i.e., those that fall between LLOD and VF. Although the ART clinical guidelines across the regions show clearly what targeted clinical interventions can be provided immediately to the patients who have been identified as VF, they have limited recommendations for clinical management of LLV cases. In addition, they define thresholds for virological failure differently. For example, the World Health Organization (WHO) definition of VF requires a VL of ≥1,000 copies/mL, but it also flags that VLs below 1,000 copies/mL typically suggest the risks for subsequent VF (7). China's National Manual for Free HIV Antiviral Treatment defines VF as VLs≥400 copies/mL, but requires no medical interventions unless the VL level is ≥1,000 copies/mL (8). In the United States, the Department of Health and Human Services (DHHS) guidelines state that patients with VL level lower than the LLOD could hardly develop drug resistance, and transient viral load increases don't suggest subsequent treatment failures, and what remains controversial is the clinical implication of LLV regarding the LLOD of 200 copies/mL, what is agreed with certainty is the ongoing process of viral evolution and accumulating drug resistance mutations even when VL is >200 copies/mL or >500 copies/mL in particular, which made sense of DHHS's threshold for VF at 200 copies/mL (9). The European AIDS Clinical Society (EACS) defines VF as VL ≥ 200 copies/mL, but it hardly provided any details to show how they have determined the virologic threshold to switch ART regimen (10). The above-mentioned HIV treatment guidelines are widely endorsed and adopted to guide clinical practice. However, they had different definitions for VF and thresholds for switches, which indicates a lack of convincing evidence regarding the common clinical phenomenon of LLV.

The existing LLV studies were mainly conducted in developed countries in regions of Europe and North America (11–13) rather than in low- and middle-income countries or regions. In South Africa, evidence from a multicenter observational cohort study that followed the WHO definitions pointed out that an LLV of 51–999 copies/mL was associated with subsequent VF, which can be viewed as an early warning sign for VF. The study recommends that clinical practitioners in the low- and middle-income countries and regions following the WHO guidelines should strengthen their efforts to differentiate and manage LLV properly, and the policy makers should include recommendations for clinical management of LLV in the WHO guidelines and reconsider a lower threshold for VF (14).

Yunnan, which lies in the southwest corner of China sharing border with the Golden Triangle in Southeast Asia, was a province hit hard by the HIV-1 epidemic with China's first HIV-1 cases diagnosed there (15). It has been recognized as one of the less developed provinces of the country. In 2018, the gross domestic production (GDP) per capita in Yunnan was $5,600 United States dollars, only 27.5% of that in Shanghai, which ranked Yunnan second last among all the provinces in China. The objective of this study was to examine whether LLVs at different levels and frequencies are associated with subsequent VFs and provide concrete evidence to inform policy making and better management of patients with LLV.

## Methods

### Study Design and Study Population

In this study, we conducted a multicenter, retrospective observational analysis of a large dynamic cohort of individuals enrolled between 15th April 2004 and 31st December 2018 and receiving ART at 235 HIV treatment centers in Yunnan, China. These HIV treatment centers include health care facilities at the four levels—provincial, prefecture/municipal, county/district, and township, as well as the ones in the confinement settings.

All HIV treatment centers treated and monitored patients following China's National Free HIV Antiretroviral Drug Treatment Manual (Version 1–4). The first-line regimens consist of 2 nucleoside reverse-transcriptase inhibitors (NRTIs) and one non-nucleoside reverse-transcriptase inhibitor (NNRTI), and the second-line regimens include two NRTIs and a protease inhibitor (PI). See Supporting Information Section—S2 for details.

A total of 110,413 subjects ever enrolled in HIV treatment between April 15, 2004 and December 31, 2018 were screened, with 24,345 cases ruled out according to the following exclusion criteria: (1) 224 subjects who were initiated on treatment at an age of <15 or with unknown dates of birth; (2) 22,002 subjects who did not have any VL measurement; (3) 1,340 subjects who did not have any VL measurement 20 weeks after treatment initiation of the respective ART regimens; (4) 779 subjects whose initial therapy was either unknown or not in compliance with the standards, such as monotherapy, two-drug therapy, three-drug therapy with wrong combination, or any ART regimens not covered by the National Manual for Free HIV Antiviral Treatment for which patients had to pay out of pocket. In total, 86,068 subjects were included in the analysis.

Recommended by China's National Free HIV Antiretroviral Drug Treatment Manual, the PI Lopinavir/Ritonavir (LPV/r) has been consistently used over the past years to replace the NNRTI for the switch to the second-line ART regimen (see Supporting Information—S2). For this study, based on the determination of whether LPV/r was used when VL tests were performed, the subjects were divided into two groups by the type of ART regimen for observation: (1) the first-line regimen group was all NNRTI-based (2 NRTIs + 1 NNRTI), with at least one documented VL measurement 20 weeks after ART initiation; (2) the second-line regimen group was PI-based (2 NRTIs + 1 PI), with at least one documented VL measurement 20 weeks after ART initiation. In case treatment for a subject was modified from the first-line regimen to the second-line therapy, the same subject was included in both groups for observational analysis of the respective courses of treatment, as long as the case has met the inclusion criteria for sub-cohorts.

### Data Collection

History cards of HIV patients as of December 31, 2018 were retrieved from the HIV/AIDS Comprehensive Response Information Management System (CRIMS) of the Chinese Center for Disease Control and Prevention. CRIMS database is real-world clinical data uploaded by all HIV treatment centers within 5 days following patient visits and is audited for data quality once a year.

### Method for VL Measurement

According to the national policy for free ART, each HIV patient can receive one VL test for free each calendar year, which means that a person with HIV will be tested free for VL at a visit ≥ 20 weeks after ART initiation and repeat the VL test once for each subsequent year. In reality, in addition to the single VL test provided free by the national program, additional VL tests may be ordered at the discretion of HIV care providers at each site with the testing cost covered by patients out of pocket or other funding resources. In case the time interval between two consecutive VL tests performed was <48 weeks, some patients may have more than one documented VL result for every 48 weeks of staying on ART. In this study, VL testing was to detect the numbers of HIV-1 RNA nucleic acid in peripheral blood plasma. VL testing was performed in the laboratories of designated HIV treatment centers at the provincial and prefecture levels. Blood samples collected by the county and township-level HIV treatment centers were separated, sub-packaged, frozen and transported to the laboratories of the prefecture-level HIV treatment centers for VL testing.

Over time, the sensitivities of the VL assays in Yunnan have been improved. Since the inception of the cohort in 2004, the HIV laboratories in the province mainly used the COBAS AmpliPrep & COBAS TaqMan 48/96 (Roche Molecular Diagnostics), the VERSANT 340/440 Molecular System bDNA (SIEMENS), and the NucliSens EasyQ Analyzer (bioMérieux) for VL assays assay. Prior to 2011, most assays used in the province had a threshold of detection that is <400 copies/mL, and a small number of assays for research or epidemiological surveillance had a higher VL sensitivity of <50 copies/mL. After that, VL assays in Yunnan were gradually upgraded with a lower detection limit of <50 copies/mL or <20 copies/mL. For All VLs, the results that were below the LLOD or had “Target Not Detected (TND)” displayed were recorded as 0 and all the quantifiable VL measurements were recorded as numerical values.

### Definitions of Key Study Variables

In this study, the start points of the two ART cohorts for observation were the dates of treatment initiation, i.e., the specific dates when the study participants were initiated on the first-line ART for the first-line ART group and the dates when LPV/r was first used in treatment for the second-line ART group. The primary endpoints for the two ART groups were when the study participants experienced VF. This study defined VF as having a first VL of ≥1,000 copies/mL at a visit ≥20 weeks after ART initiation or when switching to a LPV/r-based regimen. For the first-line ART group, the secondary endpoint refers to a patient switching to the protease inhibitor LPV/r-based regimen.

The event for observation was occurrence of LLV. In this study, the subjects who had at least one HIV VL measurement between 50 and 999 copies/mL at visits ≥20 weeks after ART initiation were defined as LLV. DHHS, EACS, China's National Manual for Free HIV Antiviral Treatment, and WHO used different thresholds to define VF, i.e., >200 copies/mL, 400 copies/mL and 1,000 copies/mL (7–10). For further analysis, this study divided LLV into three groups of HIV RNA levels accordingly, namely 50–199 copies/mL, 200–399 copies/mL, and 400–999 copies/mL. The subjects with multiple LLVs were grouped by the VL result with the highest numerical value. The subjects were also grouped as per frequency of LLV occurrence: a single episode, two consecutive episodes, two intermittent episodes, more than two consecutive episodes, and more than two intermittent episodes (15). In this study, intermittent LLV refers to an independent LLV with a previous and subsequent VL of <50 copies/mL and consecutive episodes of LLV is defined as two or more consecutive VL between 50 and 999 copies/mL.

The incidence rate for LLV was defined as the number of the subjects who had at least one episode of LLV in the study participants provided with the first-line or second-line ART regimen over the total observation time, and then stratified by calendar or follow-up year. The incidence rate for VF was calculated as the number of patients with VF, among the study participants provided with the first-line or second-line ART regimen over the total observation time, and was stratified by calendar or follow-up year.

### Statistical Analysis

The history cards of the subjects were loaded into the R3.5.0 program. The unique ART codes for HIV patients that contain no personally identifiable information were used as the primary key for data analysis. Demographic characteristics and VL measurements of the subjects are presented using descriptive statistics, with standard deviations for normal distribution and medians and interquartile ranges (IQR) for non-normal distribution, respectively. In terms of group comparisons, for continuous variables, a Student's *t*-test and Kruskai-Wallis rank sum test were used for normal and non-normal distributions, respectively after a normality test; and χ2 test was applied for categorical variables. Test level α = 0.05; for the calculation of the incidences of LLV and VF by follow-up year, the person-year incidence were reported with their 95% confidence intervals (CIs). In order to avoid bias by repeated testing in the same follow-up year, the first VL measurement of the year was used for analysis; and the multiple LLV episodes taking place in the same natural year were all included for the calculation of the natural-year incidence rate for LLV; After validation, the Cox proportional hazards model was used to assess the effect of LLVs on outcome events. The Cox analyses included the subjects who had been followed up for at least 48 weeks without experiencing VF during the same period and had two or more documented LV measurements, and excluded those who had VF for the initial VL measurement from analysis. At the time points of a subject experiencing VF or switch to second-line regimens with LPV/r, or the last VL measurement before the end of the observation point, right censoring was applied; Kaplan-Meier curves were used to show the effects of different values and frequencies of LLV occurrence on outcomes events.

## Results

**Figure 1:**
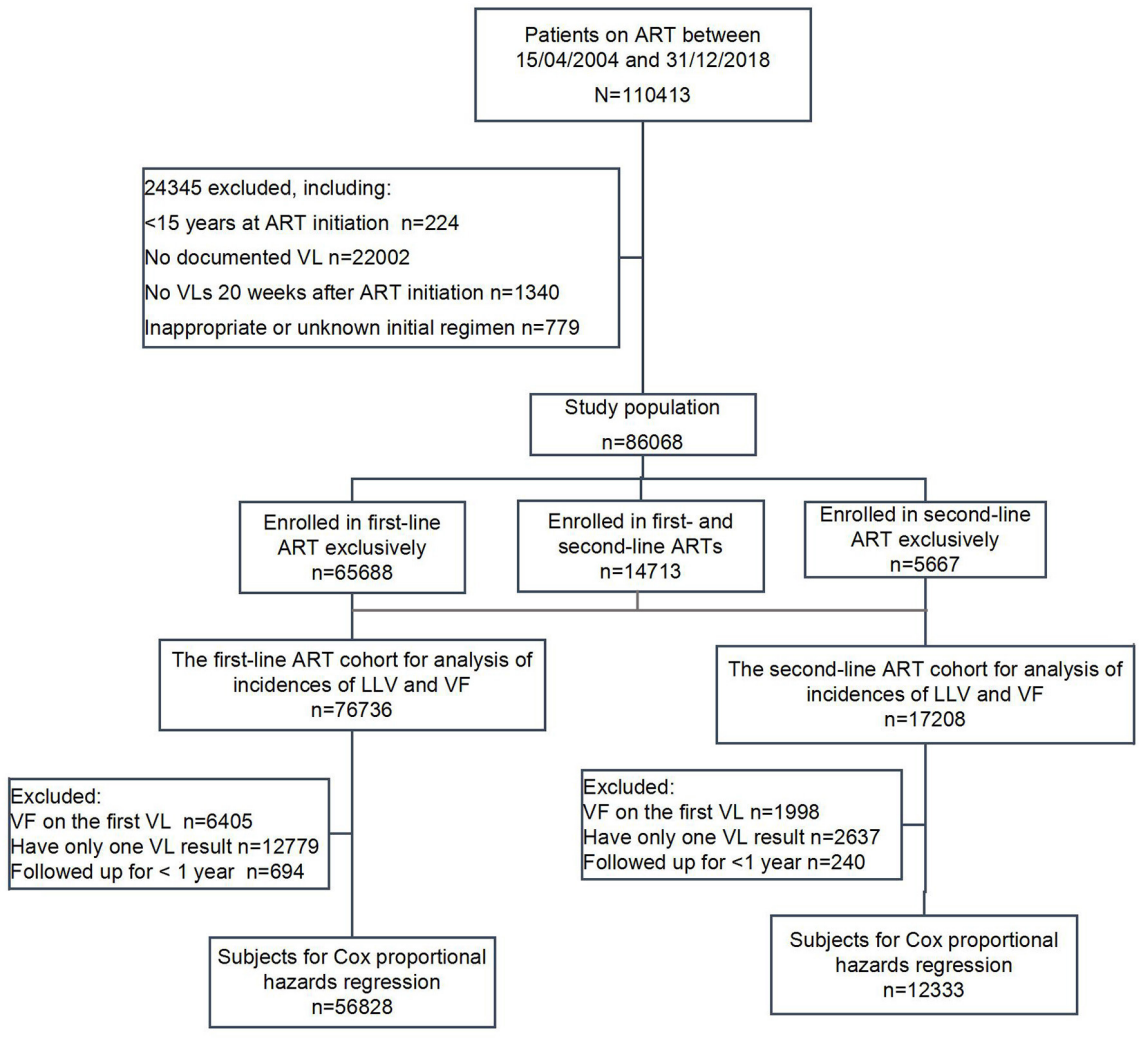
Flowchart of patient selection for data analysis.

Among the 86,068 subjects included in the analysis, 65,688 received the first-line ART (with 191,528 person-years of patient follow-up), 5,667 received second-line ART (with 11,846 person-years of patient follow-up), and 14,713 received not only first-line but also second-line ART (with 28,836 person-years of patient follow-up). Of the 14,713 subjects who received both types of ART, the individuals who met the inclusion criteria were put into the two respective ART groups for their corresponding stages of the HIV treatment, i.e., 11,048 subjects for the first-line ART group and 11,541 for the second-line ART group. In total, there were 76,736 subjects in the first-line group and 17,208 subjects in the second-line group (Figure 1).

### Demographic Characteristics and Number of VL Measurement

In the first-line ART group, male subjects outnumber females. In the second-line ART group, female subjects outnumber males. Both groups were dominated by individuals who were married or living with a partner. The majority of the subjects in the two groups were associated with heterosexual transmission. Compared with the first-line ART group, patients in the second-line ART group tended to have initiated on treatment at a younger age (37 years vs. 34 years) and with a higher CD4 count at treatment initiation (247 cells/mL vs. 253 cells/mL), and have experienced a higher CD4 increase after ART (166/mL vs. 204/mL). The median follow-up was 184 weeks (IQR 94–305 weeks) for the first-line ART group, and 156 weeks (IQR 78–256 weeks) for the second-line ART group (Table 1).

**Table 1:** Demographic characteristics of the enrolled subjects and numbers of VL tests.

**Characteristics**	**First-line ART (*n =* 76,736)**	**Second-line ART (*n =* 17,208)**
**Gender** (χ2 = 2,257 *p* < 0.001)		
Male	48,785 (63.6%)	7,561 (43.9%)
Female	27,951 (36.4%)	9,647 (56.1%)
**Marital status** (χ2 = 199.58 *p* < 0.00)		
Unmarried	14,583 (19.0%)	2,850 (16.6%)
Married or living together	50,605 (65.9%)	12,273 (71.3%)
Divorced or separated	7,131 (9.3%)	1,310 (7.6%)
Widow	4,072 (5.3%)	741 (4.3%)
Unknown	345 (0.4%)	34 (0.2%)
**Age at treatment initiation** ^*^(H = 1,321.4 *p* < 0.001)	37 [30, 46]	34 [27, 42]
**CD4 count at ART initiation** (cells/mL) ^*^(H = 7.97 *p* = 0.005)	247 [142, 350]	253 [135, 370]
**CD4 count increase** (cells/mL)^*^ (H = 311.44 *p* < 0.001)	166 [51, 313]	204 [67, 366]
**Route of HIV acquisition** (χ2 = 1856.9 *p* < 0.001)		
Intravenous drug use	13,227 (17.2%)	2,946 (17.1%)
Male-to-male intercourse	3,019 (3.9%)	357 (2.1%)
Heterosexual intercourse	55,890 (72.8%)	13,023 (75.7%)
Others (primarily includes unknown)	4,599 (6.0%)	882 (5.1%)
**Follow-up (weeks)** ^*^(H = 889.71 *p* < 0.001)	184 [94, 305]	156 [78, 256]
**Follow-up (years)** (χ^2^ = 686.86 *p* < 0.001)		
<1 year	8,474 (11.0%)	2,599 (15.1%)
1–3 years	24,407 (31.8%)	6,053 (35.2%)
3–5 years	18,395 (24.0%)	4,501 (26.2%)
>5 years	25,460 (33.2%)	4,055 (23.6%)
**Year of treatment initiation** (χ^2^ = 3197.7 *p* < 0.001)		
<2012	22,136 (28.8%)	1,421 (8.3%)
2012–2015	35,839 (46.7%)	10,040 (58.3%)
≥2016	18,761 (24.4%)	5,747 (33.4%)
**Median number of VL tests (IQR)**	3.00 [1.75, 6.00]	3.00 [1.00, 5.00]
**The maximum number of VL tests**	23	16

* *For group comparison, Kruskal-Wallis test was used for non-normal distribution after normality test*.

The median number of VL testing performed for each subject was 3 times for the two groups. The maximum number of VL tests was 23 for the first-line ART group and 16 for the second-line ART group (Table 1).

### LLV Occurrence

#### Overall Situation of LLV and VF

A total of 21,203 LLV cases were identified in the two ART groups. Specifically, 23.2% (17,832/76,736) of the subjects in the first-line group and 19.6% (3,371/17,208) of those in the second-line groups developed LLVs. Among the LLV cases, 26.4% for the first-line group (4,713/17,832) and 20.4% for the second-line group (688/3,371) experienced two or more episodes of LLV, respectively. The 76,736 subjects of the first-line ART group were followed for a total of 316917.9 person-years, with an LLV incidence of 6.63 per 100 person-years (95% CI, 5.78/100 person-years−6.17/100 person-years); the 17,208 subjects of the second-line ART group were followed for a total of 56,446.64 person-years, with an LLV incidence of 5.97 per 100 person-years (95% CI, 5.78/100 person-years−6.17/100 person-years). On the numerical level, the majority of the LLV cases had an LLV that was between 51 and 199 copies/mL (59.1% in the first-line group vs. 60.3% in the second-line group), followed by the subjects with an LLV of 400–999 copies/mL (21.8% in the first-line group vs. 19.9% in the second-line group) and the ones having an LLV of 200–399 copies/mL (19.1% in the first-line group vs. 19.8% in the second-line group); in terms of frequency of LLV, most of the LLV cases were found having a single episode (73.6% in the first-line group vs. 79.6% in the second-line group), and the proportions of LLV subjects experiencing two consecutive episodes, two intermittent episodes, more than two consecutive episodes, and more than two intermittent episodes declined sequentially (Table 2).

**Table 2:** Categorization of subjects by LLV and VF.

**Characteristics**			**First-line ART (*n =* 76,736)**	**Second-line ART (*n =* 17,208)**
	**LLV**		17,832 (23.2%)	3,371 (19.6%)
	**Categorization by numeric level**			
		50–199 copies/mL	10,542 (59.1%)	2,034 (60.3%)
		200–399 copies/mL	3,413 (19.1%)	666 (19.8%)
		400–999 copies/mL	3,877 (21.8%)	671 (19.9%)
	**Categorization by frequency of LLV**			
		A single episode	13,119 (73.6%)	2,683 (79.6%)
		Two consecutive episodes	1,891 (10.6%)	347 (10.3%)
		Two intermittent episodes	1,670 (9.4%)	217 (6.4%)
		More than two consecutive episodes	557 (3.1%)	71 (2.1%)
		More than two intermittent episodes	595 (3.3%)	53 (1.6%)
	**VF**		12,854 (16.8%)	3,304(19.2%)

Overall, 17.2% (16,158) of the subjects (*n* = 93,944) of the two ART groups experienced VF with at least one VL of ≥1,000 copies/mL. Specifically, 12,854 subjects (16.8%) in the first-line group experienced VF, and 7,196 (56%) of them were identified on their first VL measurement when they failed, and the remaining 5,658 (44%) on the subsequent VL measurement. In contrast, 3,304 subjects (19.2%) in the second-line group developed VF, with 2,085 (63.1%) and 1,219 (36.9%) subjects found on their first and subsequent VL measurement, respectively (Table 2).

#### LLVs and VFs by Follow-Up Year

In the first- and second-line ART groups, the incidence of LLV and VF was highest in the first year of ART initiation and declined steadily in the subsequent years.

**Figure 2:**
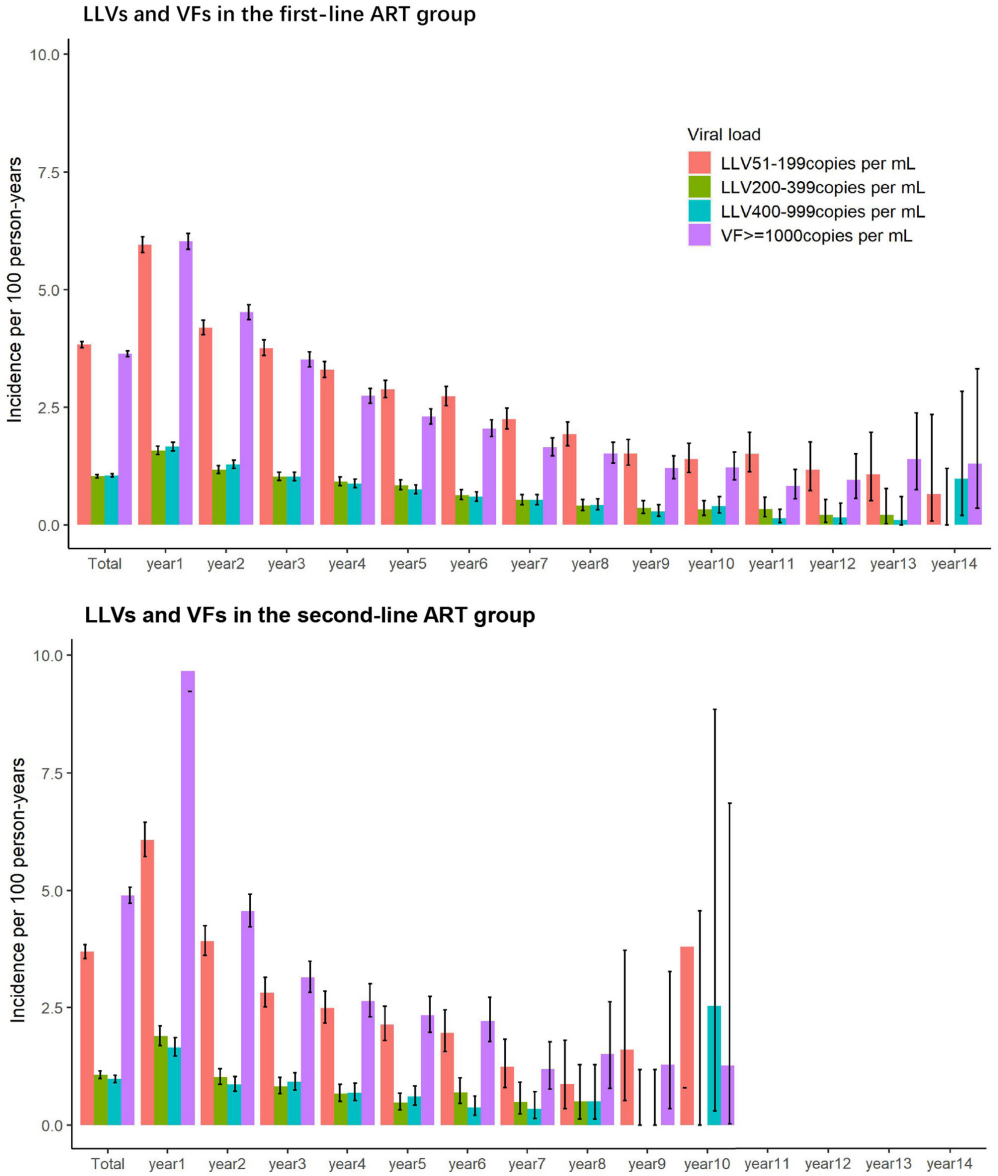
Incidence of LLV and VF by follow-up year in the two ART groups.

As illustrated in Figure 2, the incidences of LLVs of 51–199 copies/mL, 200–399 copies/mL, and 400–999 copies/mL for the first-line ART group over the first year of the first-line ART were 5.96 per 100 person-years, 1.58 per 100 person-years, and 1.67 per 100 person-years, respectively. The incidence of VF of ≥1,000 copies/mL over the first year of the treatment was 6.03 per 100 person-years. The incidences declined over the subsequent years.

A similar trend was seen with the second-line ART group. Over the first year of HIV treatment, the incidences of LLVs of 51–199 copies/mL, 200–399 copies/mL, and 400–999 copies/mL were 6.08 per 100 person-years, 1.89 per 100 person-years, and 1.65 per 100 person-years, respectively, and the incidence of VF of ≥1,000 copies/mL was 9.67 per 100 person-years. The rates decreased over the following years.

#### LLVs and VFs by Calendar Year

**Figure 3:**
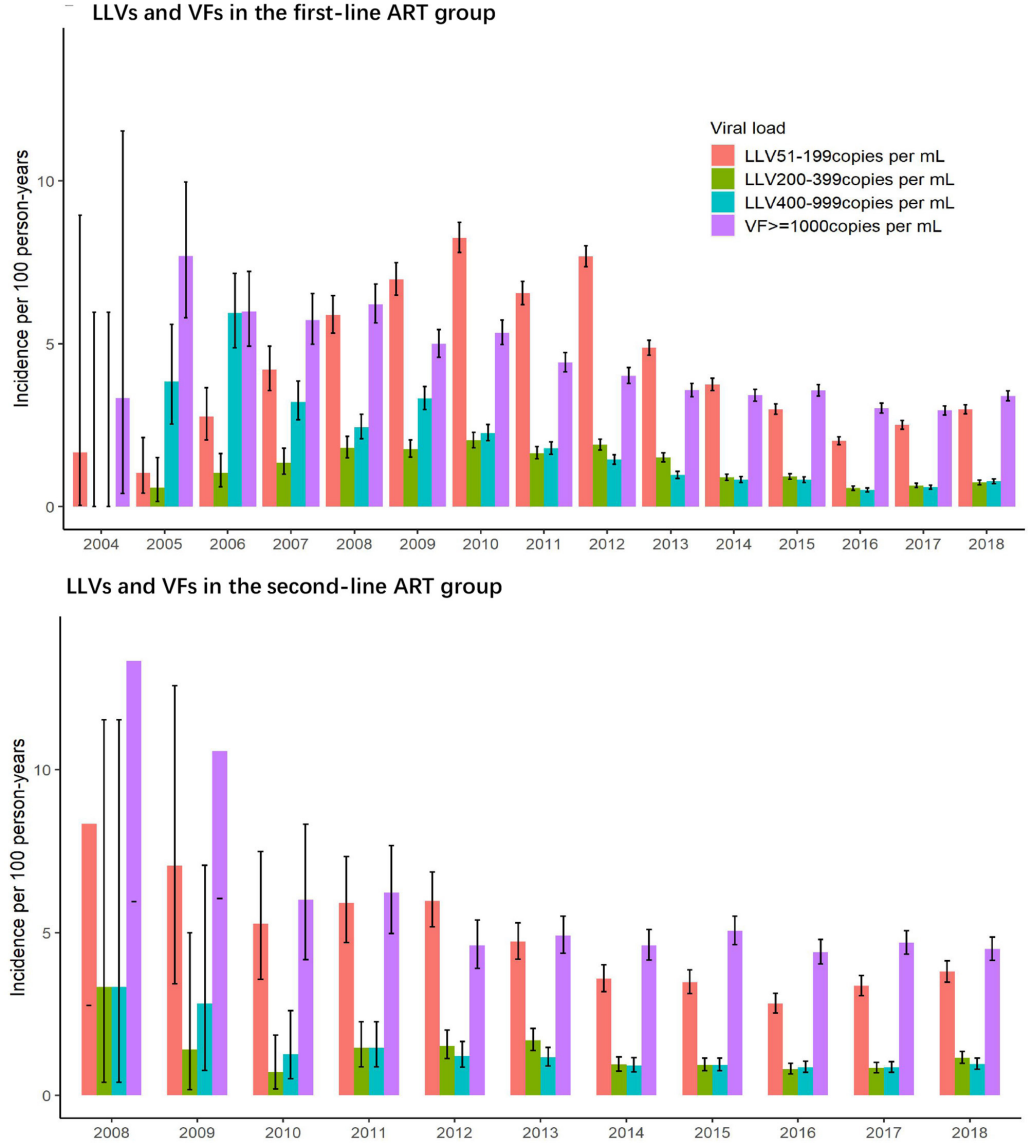
Incidence of LLV and VF by calendar year in the two ART groups.

As illustrated in Figure 3, incidences of LLV of 51–199 copies/mL and 200–399 copies/mL in the first-line group have the same trend, first reaching the peak in 2010, then decreasing to the lowest point in 2016, and climbing up again in 2017 and 2018. The incidence of LLV of 400–999 copies/mL and the incidence of VF of ≥1,000 copies/mL were similar in trend, and they decreased annually before 2012, and then stabilized over the subsequent years. The incidences of LLV by four VL levels in the second-line ART group experienced the same changes, with a decreasing trend between 2008 and 2012 and reaching a plateau afterwards.

#### Subsequent VFs After LLV

In the first-line ART group, the incidence of VF without LLV was 1.82 per 100 person-years (95% CI 1.76–1.88), whereas the incidence of VF among the subjects with LLVs of 51–999 copies/mL increased up to 2.49 per 100 person-years (95% CI 2.39–2.58). The subjects whose LLVs were between 51 and 199 copies/mL had an incidence of 1.77 per 100 person-years, which remained the same level as those who had never experienced LLV. For the subjects whose LLVs were between 200 and 399 copies/mL or between 400 and 999 copies/mL, the incidences of VF reached 2.79 per 100 person-years (95% CI. 2.56–3.03) and 4.12 per 100 person-years (95% CI 3.86–4.38), respectively.

In the second-line ART group, the incidence of VF was 2.13/100 person-years (95% CI 1.99–2.27) for the subjects without LLVs, and increased up to 2.90/100 person-years (95% CI 2.63–3.19) for those whose LLVs were between 51 and 999 copies/mL. Specifically, the incidence of VF was 2.14/100 person-years (95% CI 1.85–2.47) among the subjects with LLVs of 51–199 copies/mL, which stayed the same level as the rate for the ones who had never developed LLV. In contrast, significantly increased incidences of VF were observed among the two groups whose LLVs were either between 200 and 399 copies/mL or between 400 and 999 copies/mL, i.e., 3.16 per 100 person-years (95% CI 2.54–3.89) and 5.04 per 100 person-years (95% CI 4.24–5.94), respectively (Table 3).

**Table 3:** VFs stratified by HIV RNA levels for LLV.

**LLV by HIV RNA level**	**Number of patients**	**Person-years for** **follow-up**	**Number of VFs**	**Incidence of VF with** **95%CI (/100** **person-years)**
**First-line ART group**				
No LLV developed	52,499	212,954	3,875	1.82 (1.76–1.88)
51–999 copies/mL	17,832	103,414	2,570	2.49 (2.39–2.58)
51–199 copies/mL	10,542	60,800	1,077	1.77 (1.67–1.88)
200–399 copies/mL	3,413	19,714	550	2.79 (2.56–3.03)
400–999 copies/mL	3,877	22,900	943	4.12 (3.86–4.38)
**Second-line ART group**				
No LLV developed	11,839	42,335	900	2.13 (1.99–2.27)
51–999 copies/mL	3,371	14,011	406	2.90 (2.63–3.19)
51–199 copies/mL	2,034	8,583	184	2.14 (1.85–2.47)
200–399 copies/mL	666	2,749	87	3.16 (2.54–3.89)
400–999 copies/mL	671	2,679	135	5.04 (4.24–5.94)

In the first-line ART group, the incidence of VF was 1.82/100 person-years (95% CI 1.76–1.88) among the subjects without LLVs. It increased gradually with the increased frequency of LLV occurrence—a single episode, two consecutive episodes, and more than two consecutive episodes, i.e., 2.61 per 100 person-years (95% CI 2.49–2.73), 2.80 per 100 person-years (95% CI 2.51–3.12), and 3.50 per 100 person-years (95% CI 2.94–4.13), respectively. For the subjects who experienced intermittent LLVs, that is, having an VL of <50 copies/mL between LLVs, the incidence of VF was similar to that in the cases without LLV, with no significant difference observed.

In the second-line ART group, the incidence of VF was 2.13 per 100 person-years (95% CI 1.99–2.27) among the subjects who had never developed LLVs. The rate was increased significantly for the cases who had experienced a single episode of LLV, two consecutive episodes and more than two consecutive episodes, i.e., 3.08 per 100 person-years (95% CI 2.76–3.42), 2.96 per 100 person-years (95% CI 2.15–3.97) and 2.93 per 100 person-years (95% CI 1.42–5.33), respectively. However, the incidence of VF in cases with intermittent LLV, that is, having an VL of <50 copies/mL between LLV episodes, was similar to that among the subjects who never experienced LLV, with no significant difference observed (Table 4).

**Table 4:** VF stratified by frequency of LLV.

**Frequency of LLV**	**Number of patients**	**Person-years for** **follow-up**	**Number of VFs**	**Incidence of VF with** **95%CI (/100** **person-years)**
**First-line ART group**				
Never	52,499	212,954	3,875	1.82 (1.76–1.88)
A single episode	13,119	69,278	1,806	2.61 (2.49–2.73)
2 consecutive episodes	1,891	11,735	329	2.80 (2.51–3.12)
2 intermittent episodes	1,670	13,240	210	1.59 (1.38–1.81)
>2 consecutive episodes	557	3,834	134	3.50 (2.94–4.13)
>2 intermittent episodes	595	5,326	91	1.71 (1.38–2.09)
**Second-line ART group**				
Never	11,839	42,335	900	2.13 (1.99–2.27)
A single episode	2,683	10,633	327	3.08 (2.76–3.42)
2 consecutive episodes	347	1,452	43	2.96 (2.15–3.97)
2 intermittent episodes	217	1,229	17	1.38 (0.81–2.21)
>2 consecutive episodes	71	341	10	2.93 (1.42–5.33)
>2 intermittent episodes	53	356	9	2.53 (1.16–4.74)

### Cox Regression Analysis for LLV and VF Occurrence

In the first-line ART group, 6,405 patients who experienced VF on the first VL test, 12,779 patients who had only one documented VL result, and 694 patients who had been followed up for <1 year were excluded. A total of 56,858 cases were included in the COX proportional hazards regression analysis of LLV and VF occurrence. In the second-line ART group, 1,998 who had VF on the first VL test, 2,637 with only one VL result, and 240 followed up for no more than 1 year were excluded, with a total of 12,333 subjects included in the COX regression analysis.

#### Stratification by HIV RNA Level for LLV

**Figure 4:**
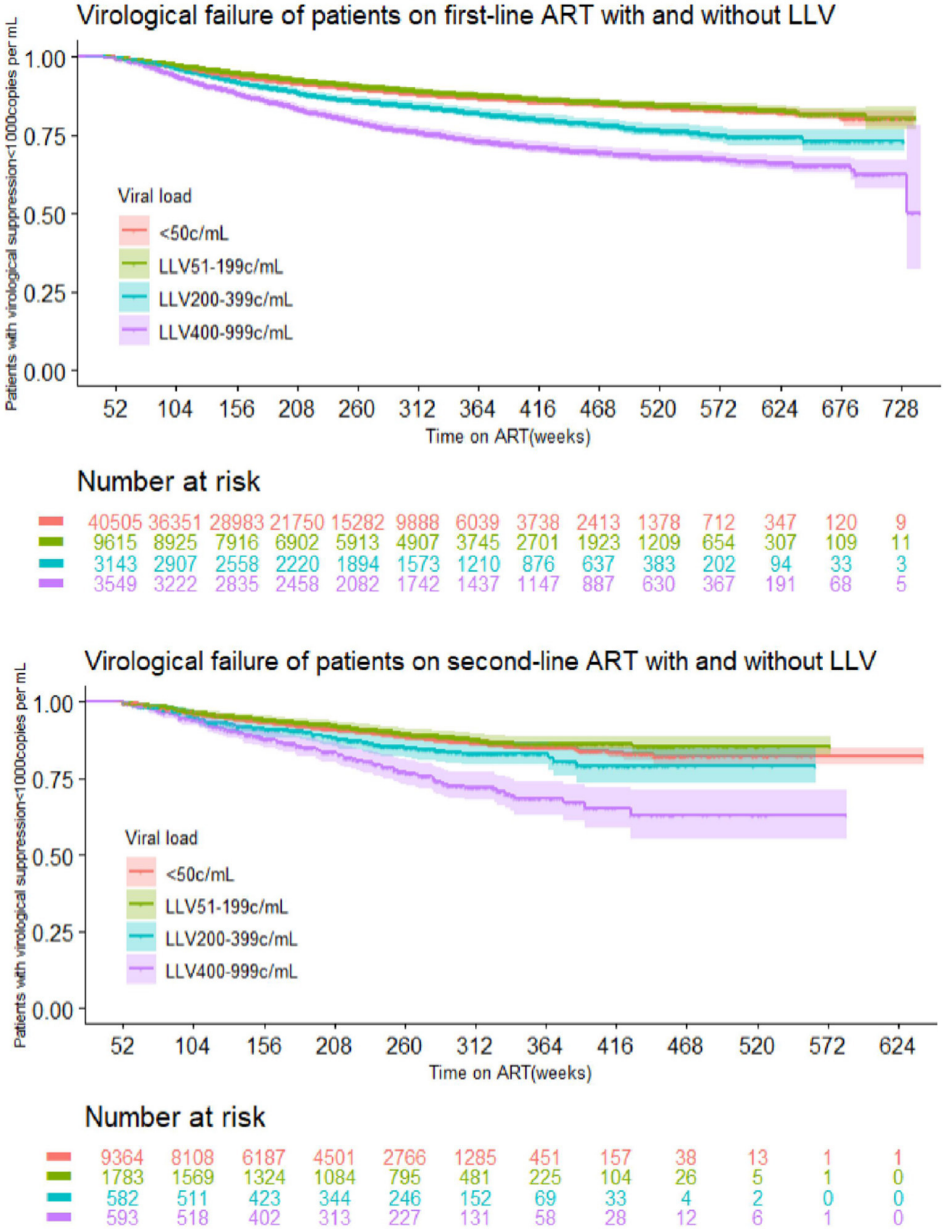
VF of patients on first- and second-line ART with and without LLV.

The multivariate COX regression analysis for LLV level and VF suggests the following results: In the first-line ART group, the risk for VF among subjects whose LLVs were between 50 and 199 copies/mL was no different from that in the cases without LLVs. The subjects with LLVs of 200–399 copies/mL and 400–999 copies/mL had a greater hazard for VF, i.e., 39 and 102% higher than the ones without LLVs, respectively. Data for the second-line ART group suggested similar trends: there was no major difference in the risk of VF between the subjects with LLVs of 50–199 copies/mL and the ones who never developed LLV, and the risk of VF in subjects with LLVs of 200–399 copies/mL and 400–999 copies/mL was 33 and 126% higher than those who had no experience of LLV (Figure 4, Table 5).

**Table 5:** Cox proportional hazards models to assess the association between LLV levels and VF.

**Variables**	**First-line ART group (*n* = 56,858)**				**Second-line ART group (*n* = 12,333)**			
	**Univariate HR**	***P*-value**	**Multivariate HR**	***P*-value**	**Univariate HR**	***P*-value**	**Multivariate HR**	***P*-value**
	**(95%CI)**		**(95%CI)**		**(95%CI)**		**(95%CI)**	
**HIV RNA level**								
LV 0–50 copies/mL	1		1		1		1	
LLV51–199 copies/mL	0.92 (0.86–0.99)	0.021	0.90 (0.84–0.97)	0.005	0.90 (0.77–1.07)	0.242	0.88 (0.74–1.05)	0.154
LLV200–399 copies/mL	1.45 (1.32–1.59)	<0.001	1.39 (1.27–1.53)	<0.001	1.34 (1.06–1.69)	0.014	1.33 (1.05–1.68)	0.019
LLV400–999 copies/mL	2.18 (2.02–2.34)	<0.001	2.02 (1.87–2.18)	<0.001	2.23 (1.85–2.69)	<0.001	2.26 (1.86–2.73)	<0.001
**Gender**								
Male	1		1		1		1	
Female	0.76 (0.72–0.80)	<0.001	0.81 (0.76–0.86)	<0.001	0.84 (0.75–0.94)	0.003	0.80 (0.69–0.92)	0.002
**Marital status**								
Unmarried	1		1		1		1	
Married/Living together	0.80 (0.75–0.86)	<0.001	0.93 (0.87–1.00)	0.048	1.00 (0.85–1.17)	0.995	1.10 (0.93–1.31)	0.269
Separated/divorced	0.88 (0.80–0.98)	0.018	1.00 (0.90–1.12)	0.969	0.97 (0.75–1.26)	0.846	1.05 (0.80–1.37)	0.726
Widow	0.72 (0.63–0.82)	<0.001	0.91 (0.79–1.05)	0.216	0.91 (0.65–1.27)	0.584	1.06 (0.74–1.51)	0.766
Unknown	0.73 (0.42–1.26)	0.257	0.85 (0.49–1.47)	0.558	1.03 (0.26–4.16)	0.964	1.10 (0.27–4.47)	0.891
**HIV acquisition category**								
Intravenous drug use	1		1		1		1	
Male-to-male intercourse	0.32 (0.26–0.40)	<0.001	0.28 (0.23–0.35)	<0.001	0.43 (0.24–0.79)	0.006	0.35 (0.19–0.65)	<0.001
Heterosexual intercourse	0.57 (0.54–0.60)	<0.001	0.61 (0.58–0.66)	<0.001	0.75 (0.65–0.86)	<0.001	0.68 (0.57–0.79)	<0.001
Others (primarily includes unknown)	0.57 (0.51–0.64)	<0.001	0.60 (0.53–0.67)	<0.001	0.68 (0.51–0.90)	0.007	0.59 (0.43–0.80)	<0.001
**Age of ART initiation**								
<30 years	1		1		1		1	
30 years−50 years	0.87 (0.81–0.91)	<0.001	0.76 (0.71–0.81)	<0.001	0.82 (0.73–0.93)	0.002	0.70 (0.61–0.80)	<0.001
>50 years	0.87 (0.80–0.94)	<0.001	0.90 (0.82–0.98)	0.017	1.10 (0.91–1.32)	0.341	1.03 (0.83–1.27)	0.818
**CD4 count at ART initiation**								
<200	1		1		1		1	
200–500	1.00 (0.95–1.06)	0.961	1.03 (0.97–1.08)	0.335	1.21 (1.07–1.37)	0.003	1.26 (1.11–1.85)	<0.001
>500	0.98 (0.95–1.06)	0.721	1.06 (0.95–1.18)	0.296	1.11 (0.91–1.35)	0.299	1.15 (0.94–1.42)	0.166
**Year of ART initiation**								
<2012	1		1		1		1	
2012–2015	0.87 (0.82–0.91)	<0.001	1.00 (0.94–1.06)	0.991	1.06 (0.89–1.25)	0.018	1.07 (0.90–1.27)	0.468
≥2016	0.79 (0.69–0.91)	<0.001	0.97 (0.84–1.11)	0.629	1.35 (1.05–1.72)	0.533	1.44 (1.12–1.85)	0.005

#### Stratification by Frequency of LLV

The multivariate COX regression models of the frequency of LLV for VF showed the following results: in the first-line ART group, compared with the patients without LLVs, the subjects who experienced a single episode of LLV (HR1.28, 95%CI 1.21–1.36, *p* < 0.001), two consecutive episodes of LLV (HR1.38, 95%CI 1.22–1.55, *p* < 0.001), and more than two consecutive episodes of LLV (HR1.76, 95%CI 1.48–2.10, *p* < 0.001) had correspondingly higher risks of VF, respectively; In the second-line ART group, the subjects who developed LLV just once (HR1.30, 95%CI 1.14–1,49), or twice in a row (HR1.36, 95%CI 1.00–1.85) were at increased risk for VF, respectively, compared with the ones who never experienced LLV (Table 6).

**Table 6:** Cox proportional hazards models to assess the association between the frequency of LLV and VF.

**Variables**	**First-line ART group (*n* = 56,858)**				**Second-line ART group (*n* = 12,333)**			
	**Univariate HR**	***P*-value**	**Multivariate HR**	***P*-value**	**Univariate HR**	***P*-value**	**Multivariate HR**	***P*-value**
	**(95%CI)**		**(95%CI)**		**(95%CI)**		**(95%CI)**	
**Frequency of LLV**								
Never	1		1		1		1	
Once only	1.33 (1.26–1.41)	<0.001	1.28 (1.21–1.36)	<0.001	1.30 (1.14–1.49)	<0.001	1.27 (1.11–1.46)	<0.001
Two consecutive episodes	1.48 (1.32–1.66)	<0.001	1.38 (1.22–1.55)	<0.001	1.36 (1.00–1.85)	0.049	1.38 (1.02–1.88)	0.039
Two intermittent episodes	0.86 (0.75–0.99)	0.035	0.80 (0.70–0.93)	0.003	0.62 (0.38–1.00)	0.051	0.63 (0.39–1.03)	0.063
>2 consecutive episodes	1.88 (1.58–2.23)	<0.001	1.76 (1.48–2.10)	<0.001	1.32 (0.71–2.46)	0.383	1.20 (0.62–2.31)	0.595
>2 intermittent episodes	0.95 (0.77–1.17)	0.624	0.85 (0.69–1.06)	0.145	1.15 (0.60–2.22)	0.676	1.25 (0.64–2.42)	0.511
**Gender**								
Male	1		1		1		1	
Female	0.76 (0.72–0.80)	<0.001	0.81 (0.76–0.86)	<0.001	0.84 (0.75–0.94)	0.003	0.80 (0.69–0.92)	0.002
**Marital status**								
Unmarried	1		1		1		1	
Married/Living together	0.80 (0.75–0.86)	<0.001	0.93 (0.87–1.00)	0.035	1.00 (0.85–1.17)	0.995	1.12 (0.94–1.33)	0.193
Separated/divorced	0.88 (0.80–0.98)	0.018	0.99 (0.89–1.10)	0.876	0.97 (0.75–1.26)	0.846	1.06 (0.81–1.39)	0.653
Widow	0.72 (0.63–0.82)	<0.001	0.92 (0.80–1.07)	0.273	0.91 (0.65–1.27)	0.584	1.08 (0.75–1.54)	0.682
Unknown	0.73 (0.42–1.26)	0.257	0.84 (0.48–1.45)	0.522	1.03 (0.26–4.16)	0.964	1.10 (0.27–1.54)	0.884
**HIV acquisition category**								
Intravenous drug use	1		1		1		1	
Male-to-male intercourse	0.32 (0.26–0.40)	<0.001	0.27 (0.22–0.34)	<0.001	0.43 (0.24–0.79)	0.006	0.34 (0.18–0.63)	<0.001
Heterosexual intercourse	0.57 (0.54–0.60)	<0.001	d0.60 (0.56–0.64)	<0.001	0.75 (0.65–0.86)	<0.001	0.67 (0.57–0.79)	<0.001
Others (primarily includes unknown)	0.57 (0.51–0.64)	<0.001	0.58 (0.51–0.65)	<0.001	0.68 (0.51–0.90)	0.007	0.58 (0.43–0.79)	<0.001
**Age of ART initiation**								
<30 years	1		1		1		1	
30 years−50 years	0.87 (0.81–0.91)	<0.001	0.75 (0.70–0.80)	<0.001	0.82 (0.73–0.93)	0.002	0.70 (0.61–0.80)	<0.001
>50 years	0.87 (0.80–0.94)	<0.001	0.89 (0.81–0.97)	0.011	1.10 (0.91–1.32)	0.341	1.01 (0.82–1.25)	0.897
**CD4 count at ART initiation**								
<200	1		1		1		1	
200–500	1.00 (0.95–1.06)	0.961	1.03 (0.97–1.08)	0.313	1.21 (1.07–1.37)	0.003	1.25 (1.10–1.42)	<0.001
>500	0.98 (0.95–1.06)	0.721	1.07 (0.96–1.19)	0.242	1.11 (0.91–1.35)	0.299	1.16 (0.95–1.42)	0.149
**Year of ART initiation**								
<2012	1		1		1		1	
2012–2015	0.87 (0.82–0.91)	<0.001	0.96 (0.90–1.02)	0.191	1.06 (0.89–1.25)	0.018	1.04 (0.87–1.24)	0.659
≥2016	0.79 (0.69–0.91)	<0.001	0.93 (0.90–1.02)	0.285	1.35 (1.05–1.72)	0.533	1.41 (1.09–1.81)	0.008

## Discussion

### The LLV Issue in HIV Antiretroviral Therapy Should Be Addressed Adequately

According to this large cohort study of HIV patients on standardized, free ART over a long period of time in Yunnan, one of the provinces with high burden of HIV where scientific responses had been put in place (16), 22.6% of the subjects developed LLV and the incidence rate of LLV over the 14 years of observation was 5.57–6.63 per 100 person-years. Compared with other studies of the same LLV definition, this result is higher than that in developed countries in the regions of Europe and North America (17, 18), and is consistent with that of the national cohort of South Africa, a member nation of BRICS (14). As of the writing of this manuscript, there were no reports on the occurrence of LLV in China's national cohort. In Shenyang, the provincial capital of Liaoning (a developed northern province of China), the incidence of LLV in a cohort of 2,155 patients enrolled in free ART by a large-size general hospital was 38.7% (19), which was apparently higher than that in this larger cohort with patients from 235 HIV treatment centers of different levels all over the less-developed Yunnan province. The exact cause of such a gap is likely to be multifactorial. For example, the incidence of LLV may vary according to different regimens and frequencies of VL testing required in different socioeconomic settings (20). In an open, single-centered, and retrospective cohort study in Beijing with 8,098 participants who were mostly HIV patients known to have infected through homosexual contact, the incidences of persistent LLV was 1.3%, lower than our study results (21).

This study indicates that, in terms of the follow-up years for the subjects, the incidences of LLV (of low, intermediate and high levels) and of VF were the highest over the first year of ART and gradually declined in the subsequent years. The yearly trend for VF in this study resonates with the findings from previous studies (22). However, the trend for occurrence of LLV was rarely studied or reported before and needs further study for validation; With regard to calendar years, the incidence of LLV for the low and intermediate levels peaked in 2010, reached the lowest point in 2016 and then rose again in 2017 and 2018. There are three main reasons for the fluctuation: (1) scaled up use of VL assays in Yunnan around 2010 with bulk procurement of equipment and increased improvement of sensitivity to lower thresholds of detection; (2) strengthened quality assurance/quality improvement (QA/QI) system for HIV care running since the founding of the ART Program Management Office in Yunnan Provincial Hospital of Infectious Disease in 2008; (3) Compromised quality of HIV care by the same number of HIV care providers due to the dramatically expanded coverage of ART program since the implementation of the ambitious plan of universal access to ART for all in 2016, with 82.5% of the diagnosed people with HIV covered by the end of 2018, which increased the accumulated number of HIV patients registered in ART up to 89,000 people, including more than 30,000 new patients enrolled over three previous years. Occurrences of high-level LLV and of VF had similar trends—decreasing on an annual basis prior to 2012 and then reaching stability afterwards. This trend suggests that high-level LLV is caused not by laboratory errors or increased precision in detection, but due to ongoing HIV replication. High-level LLV, which is simply cut off from VF by a specific threshold value, can be essentially no different from VF.

### Moderate and High Levels of LLV or Single or Consecutive Episodes of LLV Are Predictors of Virological Failure

In this study, moderate and high levels of LLV, single episodes of LLV, two consecutive episodes of LLV and more than two consecutive episodes of LLV were associated with a 1.28–2.26-fold higher risk of VF, after controlling for covariates including sex, baseline CD4 count, marital status, HIV acquisition category, age, and the year of ART initiation. A large systematic review of observation of persistent LLV in persons living with HIV reported that persistent LLV can be an independent predictor of VF and that the presence of persistent LLV should not be explained solely by variation in assay characteristics and require further investigation (22). Most of the studies using similar definitions suggested that the risk of subsequent VF was two or three times higher among the patients with persistent LLV (14, 17, 20, 22, 23). A meta-analysis of 18 ART cohorts in Europe and the United States asserted that LLV of 200–499 copies/mL was strongly associated with a 4-fold higher risk of VF (18). Like the national cohort in South Africa (14), the strength of this study was its large sample size (>70,000 subjects). Both studies had a share finding of the association between single episodes of LLV and increased hazard for subsequent VF. Some of the studies using similar definitions with sample sizes of >10,000 subjects hardly investigated the effect of a single episode of LLV on VF while the others claimed no association between them. This study concluded that 74.5% of all the subjects with LLV had single episodes. It means that HIV care providers should carefully review the HIV cases with LLV of 200–999 copies/mL and develop targeted intervention. This study also observed no significant changes with occurrence of VF after intermittent episodes of LLV and thus indicates no correlation between these two variables. A possible explanation regarding this is that intermittent LLV could be a result of laboratory variations.

### Redefining VF as Inability to Maintain a VL Below 200 Copies/mL

Increased incidence of subsequent VF in subjects with LLV of 50–199 copies/mL was not observed, indicating no association between low-level LLV and VF. In contrast, the incidence of VF was increased in subjects with intermediate- (200–399 copies/mL) and high-level (400–999 copies/mL) LLV, resulting in an increasingly higher risk of subsequent VF. This conclusion echoed with the DHHS guidelines and the EACS guidelines for defining a VL of ≥200 copies/mL as the virologic threshold for VF. As evidenced by more studies and clinical practice, it is recommended that a lower threshold VL for VF should be considered in the WHO guidelines and China's National Manual for Free HIV Antiviral Treatment in order to endorse timely medical intervention for the patients with VLs of 200–1,000 copies/mL.

### Recommendations for Clinical Management of LLV Cases in Yunnan and in High HIV-Prevalence Settings

**Figure 5:**
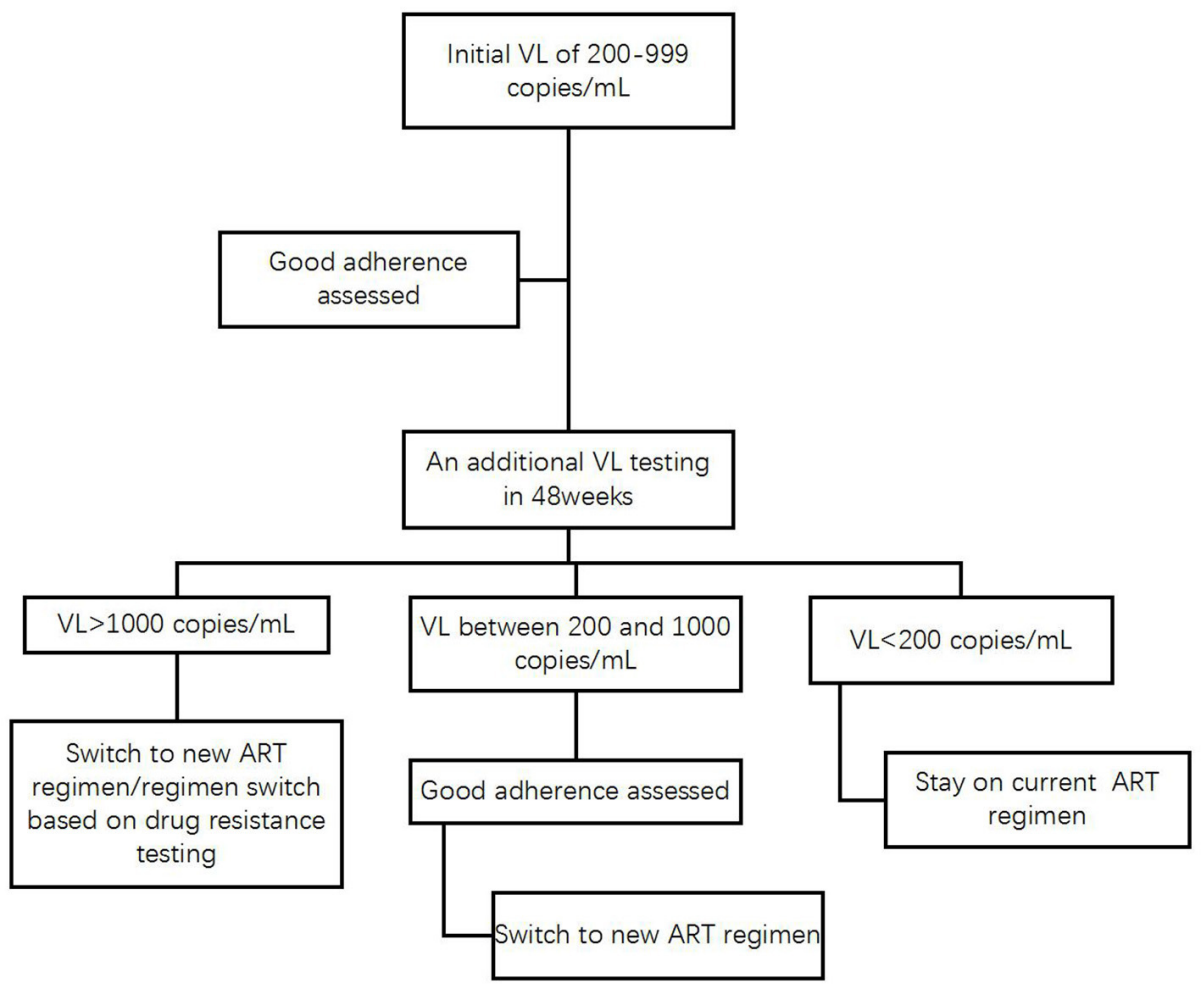
Recommended algorithm for management of HIV patients with LLV in Yunnan.

Without waiting for the final revision of the definition of VF in the WHO guidelines and the National Free AIDS Antiretroviral Drug Treatment Manual, the HIV care providers in Yunnan and any other high-HIV-prevalence settings could give immediate medical attention to the HIV patients on ART who initially experience LLVs of 200–999 copies/mL, assess patient adherence and provide targeted education, counseling and support through HIV case managers (24). Constrained by limited HIV/AIDS resources available for the province, HIV patients in Yunnan can access free VL testing once a year and a free test to detect drug resistance in case VL is >1,000 copies/mL. It is recommended that patients should be mobilized to pay out of pocket for receiving an additional VL test in 24 weeks in order to determine if they experience intermittent or persistent LLV and have the virologic response monitored. In case the additional VL of a patient is >1,000 copies/mL or remains between 200 and 999 copies/mL as consecutive episodes of LLV, HIV care providers should determine VF and switch to new regimens for the patients who demonstrated good treatment adherence (Figure 5).

## Limitation

Research design of this study was focused on grouping of the subjects by HIV RNA level and frequency of LLVs. Without cross cluster analysis of these two categorical variables, we could not generate findings in clearer ways to pinpoint targeted interventions for HIV patients; This observational study, which was based on real-world data, lacked randomization and could be biased in selection and exclusion of research participants.

More than 20,000 individuals were excluded from data analysis due to the absence of documented VL testing results although there was statistical difference between the excluded subjects and the study participants in terms of their demographic characteristics. Compared with study participants, the subjects who were excluded from data analysis tended to be older in age (39.4 years vs. 37.2 years), had lower levels of CD4 count at baseline (237 copies/mL vs. 252 copies/mL), had a higher percentage of males (68.2% vs. 60.7%), and included more people with HIV enrolled in ART since 2016. Among the HIV patients initiated on ART since 2016, a large number of HIV patients were treated for <48 weeks, and had no documented VL results, constrained by the national HIV/AIDS treatment policy of one free VL testing each calendar year. The impact of missing values for the excluded subjects on the findings was unclear.

Previous studies suggested that a VL of >99,999 copies/mL at baseline was associated with a higher risk for episodes of LLV (25). In this study, the absence of baseline VLs for each patient due to the lack of financial resources for provision of free VL testing at baseline may have important implication for data analysis; findings of this study could be distorted due to confounding by a large number of individuals with varying capacities across a wide range of VL testing laboratories all over the province delivering laboratory testing services with varying levels of quality; most of the subjects were tested for VL only once a year due to limited HIV/AIDS resources, and thus this study can be compromised by limited frequency of VL testing performed for each subject; Some VL assays used in Yunnan had less sensitivity with a threshold of <400 copies/mL until 2011. Out of the 383,595 documented VL tests included for data analysis, 36,585 among 16,851 patients were performed prior to 2011. Therefore, some VL tests included in data analysis used different threshold for detection and may fail to meet the LLOD standard defined in this study. No targeted analysis was conducted in this study to understand how it may impact on the findings. The findings of this study could hardly be generalized for those who were not tested for VL because only the subjects with documented VL results were included for data analysis.

## Conclusion

The retrospective observational analysis of the large dynamic cohort of HIV ART in Yunnan province identified frequent occurrence of LLV. In conclusion, our results suggest that single or consecutive episodes of intermediate- or high-level LLV predicts increased risk for VF, which necessitated further investigation and immediate interventions accordingly. This study forms the basis for development of evidence-based management of HIV patients with LLV in Yunnan.

## Data Availability Statement

The raw data supporting the conclusions of this article will be made available by the authors, without undue reservation.

## Ethics Statement

The study was approved by the Medical Ethics Committee, Yunnan Provincial Hospital of Infectious Disease (Approved Document No. K201913). In this study, the rights and interests of the participants were fully protected, with no potential risk posed to the subjects. Written informed consent to participate in this study was provided by the participants' legal guardian/next of kin.

## Author Contributions

JA, YL, and JL designed the study and developed the research protocol and tools. JA and JL were responsible for data collection, analyzed the data, interpreted the results, and drafted the manuscript with guidance from ST, TL, and XD. ST reviewed and contributed to editing the manuscript. All authors have read and approved the final manuscript.

## Funding

This project has been supported with the Joint Fund for Applied Basic Research (No. 2020202001AY070001-105) from the Yunnan Provincial Department of Science and Technology, Kunming Medical University, the Yunnan Infectious Disease (HIV/AIDS) Clinical Trial Research Center Project (202102AA310005-031), and China Free HIV Antiretrovial Treatment Program.

## Conflict of Interest

The authors declare that the research was conducted in the absence of any commercial or financial relationships that could be construed as a potential conflict of interest.

## Publisher's Note

All claims expressed in this article are solely those of the authors and do not necessarily represent those of their affiliated organizations, or those of the publisher, the editors and the reviewers. Any product that may be evaluated in this article, or claim that may be made by its manufacturer, is not guaranteed or endorsed by the publisher.
