# Supplementary material for: The Impact of Low-Level Viraemia on Virological Failure—Results From a Multicenter HIV Antiretroviral Therapy Cohort Study in Yunnan, China
Source: Front Med (Lausanne). 2022 Jul 4;9:939261. doi: 10.3389/fmed.2022.939261 (PMC9289465; doi:10.3389/fmed.2022.939261)
Supplement: Supplementary file 1 [file Data_Sheet_1.docx]

**Appendix: The Impact of Low-Level Viraemia on Virological Failure—Results from a Multicenter HIV Antiretroviral Therapy Cohort Study in Yunnan, China**

**S1. Comparison of clinical and demographic characteristics between the excluded subjects and study participants**

| **Variables** | **Excluded subjects（n=24345)** | **Study participants (n=86068)** | **H/χ2** | **P value** |
| --- | --- | --- | --- | --- |
| Median age at ART initiation [IQR] | 39.4 [31.7, 49.3] | 37.2 [30.3, 45.8] | 568.4 | <0.001 |
| Median CD4 count at baseline [IQR] | 237 [118, 369] | 252 [145, 361] | 96.784 | <0.001 |
| Gender n (%) |  |  | 454.35 | <0.001 |
| Female | 7749 (31.8) | 33851 (39.3) |  |  |
| Male | 16596(68.2) | 52217(60.7) |  |  |
| Year of ART initiation |  |  | 8884.9 | <0.001 |
| <2012 | 4194(17.2) | 23124(26.9) |  |  |
| 2012-2015 | 6322(26.0) | 41454(48.2) |  |  |
| >=2016 | 13828(56.8) | 21490(25.0) |  |  |

Note: Normality test was run for the age at ART initiation and CD4 count at baseline, p<0.001; For group comparison, Kruskal-Wallis rank-sum test was used. Chi-square analysis was applied for qualitative data.

**S2. Recommended Standard Regimens in China’s National Manual of Free HIV Antiretroviral Treatment**

| **First-line ART** | |
| --- | --- |
| 2005 Edition | d4T+3TC+NVP |
| 2008 Edition | AZT or d4T+3TC+NVP |
| 2012 Edition | TDF or AZT+3TC+EFV or NVP |
| 2016 Edition | TDF or AZT+3TC+EFV or NVP  TDF or EFV is preferred except for contraindications |
| **Second-line ART** | |
| 2008 Edition | TDF+3TC+LPV/r |
| 2012 Edition | TDF or AZT+3TC+LPV/r |
| 2016 Edition | TDF or AZT or ABC+3TC+LPV/r  AZT+TDF+3TC+LPV/r（HIV co-infection with HBV） |
